# Retrotransposon-induced mosaicism in the neural genome

**DOI:** 10.1098/rsob.180074

**Published:** 2018-07-18

**Authors:** Gabriela O. Bodea, Eleanor G. Z. McKelvey, Geoffrey J. Faulkner

**Affiliations:** 1 Mater Research Institute—University of Queensland, TRI Building, Brisbane, Queensland 4102, Australia; 2 Queensland Brain Institute, University of Queensland, Brisbane, Queensland 4072, Australia

**Keywords:** retrotransposon, LINE-1, neuron, neurogenesis, mosaicism

## Abstract

Over the past decade, major discoveries in retrotransposon biology have depicted the neural genome as a dynamic structure during life. In particular, the retrotransposon LINE-1 (L1) has been shown to be transcribed and mobilized in the brain. Retrotransposition in the developing brain, as well as during adult neurogenesis, provides a milieu in which neural diversity can arise. Dysregulation of retrotransposon activity may also contribute to neurological disease. Here, we review recent reports of retrotransposon activity in the brain, and discuss the temporal nature of retrotransposition and its regulation in neural cells in response to stimuli. We also put forward hypotheses regarding the significance of retrotransposons for brain development and neurological function, and consider the potential implications of this phenomenon for neuropsychiatric and neurodegenerative conditions.

## 1. Introduction

The mammalian brain is remarkably complex in form and function. Neural cell diversity underpins this complexity, and has classically been defined in terms of the morphological differences between cell types, their diverse connectivity patterns, physiological and functional properties and the expression of various transcription factors (TFs), cell surface and secreted molecules [1,2]. At the same time, it has been assumed that the neural cells of any individual will all carry the same genetic instructions, or genotype, and that cellular diversity is achieved by changing how these instructions are read, for example, through epigenetic modifications [3]. However, recent advances in DNA sequencing and genetic analysis, as well as bioinformatics, have made it possible to identify mutations generating distinct genotypes among the neurons of an individual human brain. These mutations include single-nucleotide variants, copy number variants (CNVs) and retrotransposon insertions [4–10]. Collectively, these genetic variants form a landscape of somatic mosaicism within the brain.

Somatic mosaicism is defined as the existence of two or more cells with different genotypes within one individual. Depending on its developmental timing, mosaicism can encompass mutations that are heritable (germline mosaicism), non-heritable (somatic mosaicism) or a combination of these two outcomes [9,11–14]. Retrotransposons, colloquially referred to as ‘jumping genes’, are DNA fragments that have the ability to copy their sequences from one location in the genome and insert themselves into a new genomic position, causing mutations and changing the genomic landscape at the new integration site. It is now established that retrotransposons are endogenous mediators of somatic mosaicism in the brain [4–7,10,14]. As remnants of mammalian genome evolution, retrotransposons may occupy up to two-thirds of the human genome [15], but most human retrotransposon copies have lost their ability to mobilize further [16]. However, some elements are still capable of mobilization, in the germline and in the soma. Over the past decade, several studies have shown that retrotransposons contribute to intra-individual variation in neuronal genomes, defining a new layer of neural diversity and heterogeneity. Maintenance of this complex heterogeneity may be essential for healthy brain function, while its disruption may play a role in neurological diseases [17,18]. To provide a comprehensive understanding of the roles retrotransposons play in neural function, and their potential contribution to disease, we will first present an overview of the active retrotransposon families in humans, their mobilization mechanism, and experimental strategies for studying neuronal retrotransposition.

## 2. Types of retrotransposons in the human genome

**Figure 1.**
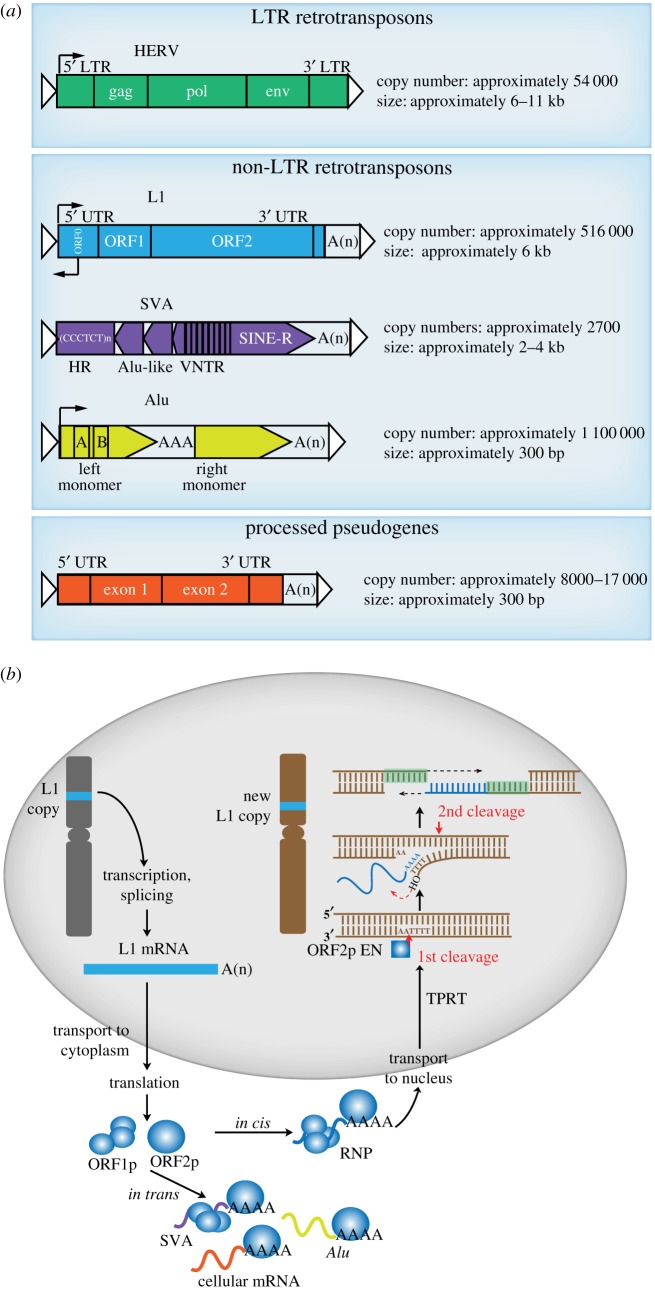
Human retrotransposon families and mobilization mechanism. (*a*) Types of retrotransposons, their mobilization capacity, size and number of copies in the human genome*.* HERV, human endogenous retroviruses consisting of two LTRs; gag, group-specific antigen; pol, polymerase; env, envelope gene; long interspersed element-1 (L1) structure: 5′ untranslated region (UTR) with open reading frame (ORF)0 and promoter activity; ORF1 and ORF2; 3′ UTR and poly(A) tail; SVA structure: CCCTCT hexamer repeats (HR), *Alu* sequence in reverse orientation (*Alu*-like); VNTR, variable number of tandem repeats; SINE-R, a short interspersed element of HERV origin; An, poly(A) tail; *Alu* structure: a left monomer with internal RNA polymerase III promoter binding sites (A, B boxes); AAA, adenosine-rich linker; right monomer ending in poly(A) tail (An); processed pseudogene: sequence derived from cellular messenger RNA, which has been reverse transcribed into DNA and has no introns; target side duplications are shown as white triangles. (*b*) L1 retrotransposition mechanism as an example of retrotransposon mobilization. A full-length, retrotransposition-competent L1 is present at one genomic locus (blue box in the left grey chromosome). The L1 encodes two proteins essential for its mobility, ORF1p (with nucleic acid chaperone activity [31,32]) and ORF2p (with endonuclease [33] and reverse transcriptase [34] activity), as well as an unusual antisense ORF0 that may facilitate retrotransposition [35]. L1 transcription results in a bicistronic, polyadenylated mRNA (blue rectangle), which is transported to the cytoplasm for translation. Upon translation, ORF1p and ORF2p (blue spheres) bind to their encoding L1 mRNA *in* *cis* and form an RNP complex. Note that the L1 proteins can retrotranspose cellular mRNAs to generate processed pseudogenes, as well as SVA and *Alu* retrotransposons (purple, red and yellow wavy lines). Once the RNP is formed, it enters the nucleus through a still poorly understood process, where a new L1 insertion occurs by target-site primed reverse transcription (TPRT) [36,37]. During TPRT, the ORF2p endonuclease makes a first and second cleavage (red arrows) in the genomic DNA at the consensus sequence 5'-TTTT/AA [33], and releases a 3' hydroxyl (OH) group from which the ORF2p reverse transcriptase initiates reverse transcription of the attached L1 mRNA (indicated by the red dashed line arrow). The DNA fragment between the two cleavages is highlighted in green to indicate the formation of TSDs. The black dashed arrow indicates completing synthesis across the second strand of cDNA, resulting in a new L1 copy, and the TSDs, which flank the new L1 copy.

Retrotransposons are the main class of transposable elements found in most mammalian genomes. Based on the presence of long terminal repeats (LTR) in their structure, they are classified into LTR and non-LTR retrotransposons. The LTR group consists of ERVs (endogenous retroviruses), which comprise approximately 8% of the human genome and represent remnant retrovirus sequences incorporated into the host germline after ancient viral infections [19,20]. The non-LTR retrotransposon group is further subdivided based on the ability of elements to mobilize independently or only with the machinery encoded by another retrotransposon, into autonomous and non-autonomous elements, respectively. Long interspersed element 1 (LINE-1 or L1) is the only autonomous non-LTR retrotransposon in humans. In addition to its own mobilization *in cis*, L1 can mobilize *in trans* non-autonomous short interspersed element (SINE) retrotransposons. These include 7SL-derived *Alu* elements, composite SVA (SINE–variable number tandem repeat–*Alu*) elements, and cellular mRNAs, resulting in processed pseudogenes [21–25]. L1 copies can be over 6 kb in length and occupy the highest proportion of the human genome by sequence of all the transposable elements (approx. 17%) [19]. *Alu* copies are approximately 300 bp in size and represent the most abundant transposable element by copy number (approx. 11% by sequence) [19,26,27]. SVAs are approximately 2 kb in size, with far fewer copies than L1 or *Alu*, and comprise only about 0.2% of the genome sequence [19,28–30] (figure 1*a*).

Owing to truncation, deletion, internal rearrangement and other mutations, the vast majority of human retrotransposon sequences are incapable of further mobilization and are considered molecular fossils [16]. Computational-, molecular- and genomics-based studies have to date identified and catalogued retrotransposons into evolutionary families and subfamilies. Only a few L1, *Alu* and SVA subfamilies remain mobile. Moreover, functional studies have identified that most retrotransposition-competent L1s are part of a small group of elements termed Ta (transcribed-active) L1s [38]. All of the L1s reported to be highly active *in vitro* to date belong to the L1-Ta and L1-preTa families [39–42]. Similarly, particular *Alu* subfamilies, such as AluYa5 and AluYb8 [43], and SVA E, F and F1 subfamilies [19,24,29] have been identified as the most active in humans. Human ERVs (HERVs) are considered to be immobile in modern humans, although a recent study revealed several polymorphic HERV-K insertions in the global population and a full-length, intact, insertion with potentially functional open reading frames (ORFs) [44]. However, it remains unclear whether this insertion is a recently integrated provirus or has simply been lost in some individuals.

## 3. An overview of the retrotransposon mobilization mechanism

All retrotransposons mobilize via a replication mechanism involving an RNA intermediate [45]. During this process, termed retrotransposition, the host polymerase starts the synthesis of an RNA copy of the element and, subsequently, the RNA is reverse transcribed into DNA by an element-encoded reverse transcriptase. HERV reverse transcription takes place in the cytoplasm and the viral DNA is then transported into the nucleus and integrated into a viral integrase which catalyses insertion of the HERV into its new target site [46]. L1 produces a bicistronic mRNA that is transported to the cytoplasm and translated. L1 encodes proteins that present a strong *cis*-preference [47], meaning they tend to bind to their encoding mRNA. This results in the formation of ribonucleoprotein particles (RNPs) that may localize to cytoplasmic stress granules [48,49] or enter the nucleus. In the case of L1, reverse transcription occurs in the nucleus, where the L1-encoded endonuclease [33] makes a single-strand cleavage at the target DNA, exposing a 3' hydroxyl group, which is used as a primer for reverse transcription by the L1-encoded reverse transcriptase [34] (termed target-primed reverse transcription, or TPRT) [36,37]. TPRT typically results in L1 insertions with the following sequence features: insertion at an L1 endonuclease motif (5'-TTTT/AA), target site duplications (TSDs) and a poly(A) tail (figure 1*b*) [33,50,51].

Retrotransposition does not always result in the faithful duplication of an element, particularly in the case of L1. Owing to errors during reverse transcription, only approximately 40% of L1 copies are identical to their source, or donor, L1 [52]. Also, 5' truncation, which is a common characteristic of recent human L1 insertions [19,53], may occur, likely because of the premature dissociation of the reverse transcriptase from the L1 mRNA strand during TPRT. The precise mechanism through which 5' truncation occurs is not clearly understood, although it has been speculated that DNA repair may play a role as host DNA repair factors, such as ATM (ataxia telangiectasia mutated), may limit the size of L1 insertions [54].

As a result of 5' truncations, internal inversions or deletions [55] and point mutations, only about 80–100 [41,42] L1 copies are estimated to be able to retrotranspose per average human genome, with even fewer copies accounting for most new L1 insertions [41,56–58]. One new L1 insertion is generated per 100–200 human births [42,59]. By contrast, 3000 L1 copies [60] are estimated to be retrotransposition-competent in the mouse genome, with at least one in eight mice harbouring a new L1 insertion [11].

## 4. Detection of retrotransposon activity in the healthy brain

Although nearly 70 years have passed since Barbara McClintock's seminal studies of transposable elements in maize [61,62], ongoing L1 retrotransposition in modern human genomes was only demonstrated in 1988 [63]. Subsequently, endogenous L1 retrotransposition has been described in early embryonic development, germ cells and various diseases, such as cancer [63–69]. Insertions arising during early development or in cancer cells are likely to undergo clonal expansion, resulting in a significant proportion of cells harbouring a particular insertion. Studying the contribution of L1 insertions to somatic mosaicism in normal tissues, however, presents a particular technical challenge, as these genomic variants may be found only in a limited number of cells.

Over the past decade, three main tools have facilitated the discovery and study of L1 retrotransposition in the brain. Firstly, an engineered L1 reporter construct, previously developed to recapitulate L1 retrotransposition in cultured cells [50,70], has been used as a functional read-out of L1 retrotransposition in human embryonic stem cell (hESC)-derived neural progenitor cells (NPCs), rodent NPC cultures and transgenic animal models [71]. The L1 construct consists of a human L1 sequence and a gene encoding enhanced green fluorescent protein (EGFP) in reverse orientation to the L1 transcript. The *EGFP* gene is interrupted by an intron in the same transcriptional orientation as the L1. Thus, only cells with successful L1 retrotransposition events can express GFP because the EGFP intron is removed from the RNA intermediate, followed by reverse transcription and integration into a genomic location. When the L1-EGFP system is employed in neuronal cultures, GFP co-localizes with neuronal markers Map2 and β-tubulin, whereas very little to no GFP co-localizes with astrocyte or oligodendrocyte markers [72]. Neurons obtained from transgenic L1-EGFP mouse brain sections were positive for GFP expression, in a variety of different brain areas, such as cortex, hypothalamus, amygdala and hippocampus [72–74]. Although the genomic location of the engineered L1 transgene may result in unknown epigenetic effects that do not necessarily recapitulate the regulation of endogenous L1 copies, this type of assay remains a valid tool to understand the effects of L1 and other retrotransposon activity on neural cell function. With recent advances in genome editing technology [75], the design of site-targeted reporter constructs, as well as conditional inducible constructs for temporal and cell-specificity, is more easily achievable and may be instrumental in dissecting the parameters of retrotransposition in the brain.

Secondly, to estimate the number of L1 copies across different tissues and brain areas, Coufal *et al*. [76] developed a TaqMan quantitative PCR-based L1 CNV assay. This assay estimated an enrichment of L1 copies in the human brain when compared with other tissues, with more L1 copies also found in the hippocampal dentate gyrus than in other brain areas [4,74,76]. The authors [76] estimated 80 somatic L1 insertions occurred per neuron. Owing to the high copy numbers of L1 sequences already present in the genome [19], this assay offers very limited sensitivity when attempting to quantify variation in the number of L1 insertions in somatic tissues. Moreover, the assay does not discriminate integrated copies generated by TPRT from potential extrachromosomal L1 DNAs or any other source of L1 CNV not arising from retrotransposition [77]. New quantitative PCR approaches, such as digital droplet PCR (ddPCR), may prove a better tool to quantify retrotransposon CNV in a more accurate but still cost-effective manner [78,79].

Thirdly, high-throughput DNA sequencing strategies have been developed to detect, quantify and resolve L1 insertions. Whole-genome sequencing (WGS) or targeted sequencing of retrotransposon–genome junctions has been applied to bulk brain, pooled neurons and whole-genome amplified (WGA) material from single neuronal nuclei from post-mortem human brain [4–7,10,80]. Tissues from other parts of the body, such as liver or blood, have been used to discriminate somatic and polymorphic insertions (i.e. insertions found in neurons and absent in non-brain tissue are annotated as somatic). Databases of known retrotransposon insertion polymorphisms can also be used to discriminate novel and known insertions. To fully characterize most new insertions, thorough PCR validation followed by Sanger sequencing is required [81,82]. One of the advantages of using high-throughput DNA sequencing is that it allows not only for detection and quantification of endogenous L1 variants, but also for resolving the structure and genomic location of these insertions. Resolving the structure of a specific insertion is paramount in understanding the potential functional impact of the insertion on the genome.

Providing a valuable orthogonal approach to single-cell genomic analysis, a recent study used somatic cell nuclear transfer to reprogramme postmitotic olfactory bulb neurons into enucleated oocytes and cloned the neuronal genome to produce enough DNA for WGS without the need for WGA. WGS analysis of the resulting clones revealed several de novo L1 insertions [14]. These studies altogether demonstrate that L1-driven somatic mosaicism can occur in the mammalian brain. However, with single-cell genomic analysis in its infancy, methodological variation, including the use of different cell populations as starting material, has led to estimates ranging from one L1 insertion per 25 neurons [5] to 13.7 L1 insertions per neuron [10]. Therefore, although these studies offer strong evidence of L1 retrotransposition in the brain, the frequency of retrotransposition in neural cells requires further investigation [83].

## 5. Is there a temporal niche for retrotransposition in the brain?

**Figure 2.**
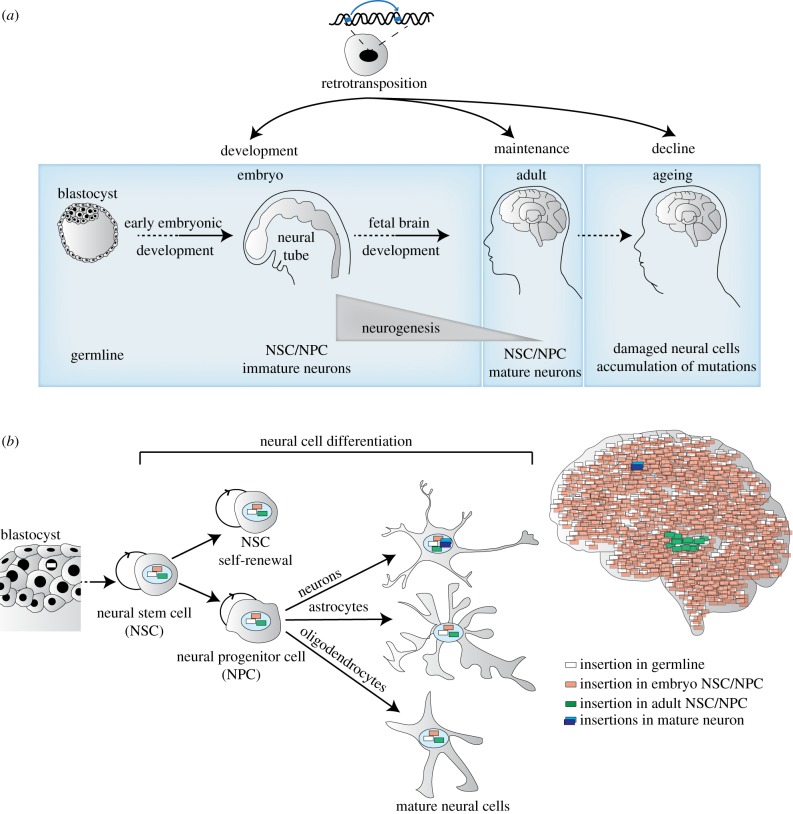
Timeline of retrotransposition activity in the brain. (*a*) Retrotransposition can occur in the germline, during embryogenesis, as well as during nervous system development, and can involve neural stem cells (NSCs), NPCs and post-mitotic neurons. Retrotransposition events could accumulate over time and also, potentially, take place later in life as a consequence of the ageing process. (*b*) Depending on its timing and cell type, a single retrotransposition event can become clonally expanded in the neural cells of the adult brain, with potentially significant effects on brain physiology. When retrotransposition occurs later in brain development, or even in post-mitotic neural cells, new insertions are restricted to one cell, which can in turn be selected against during development or maintained in adults but with a lesser probability of affecting overall brain function.

Retrotransposition in the brain could occur at any stage of life, from early brain development to brain maintenance and decline during adulthood, and also influence neural diversity and survival (figure 2*a*). Neural diversity arises during early embryogenesis through the spatial and temporal patterning of neuroblasts and neuronal progenitor cells. A complex network of gene expression determines a particular cell type-specific progenitor domain and fate [84]. Expression of pan-neuronal genes marks the beginning of neurogenesis and specific TFs are required for neuronal differentiation. Differentiating neurons migrate, grow axonal processes and later form synapses. Various signalling molecules, TFs and cell receptor molecules tightly control this development [85]. Although the bulk of neurons are generated during embryonic neurogenesis, neurons are also born in specific areas of the postnatal brain, such as hippocampus, olfactory system and amygdala [86,87]. An estimated 700 neurons are renewed in the human hippocampus daily [88]. Whether retrotransposition occurs during adult neurogenesis, or during embryonic development, in neuroblasts, committed neural progenitors, or in mature neural cells, determines the number of somatic L1 insertions harboured by a given cell. The spatio-temporal parameters of somatic L1 retrotransposition therefore will likely determine the number of cells that are mosaic for a particular insertion and, most importantly, will govern the overall impact an individual insertion may have on brain activity (figure 2*b*).

### 5.1. Retrotransposition in embryonic and adult neurogenesis

Over a decade ago, Muotri *et al*. [72] presented the first evidence that L1 retrotransposons can mobilize during neuronal differentiation. Using a human L1-EGFP reporter system, L1 expression was observed both *in vitro*, in rodent primary hippocampus-derived NPC cultures, and *in vivo*, in transgenic mice. L1 expression in transgenic mice was observed as early as embryonic day (E) 12.5, when the first waves of immature differentiated neurons appear in the brain [72]. These data were later corroborated and advanced by Coufal *et al*. [76] in a study reporting L1 reporter activity and endogenous L1 mRNA expression in human NPCs either isolated from fetal brain or derived from hESCs. The preference of retrotransposition for proliferating progenitor cells may be related to the cell cycle. Several studies reported an elevated rate of L1 retrotransposition in dividing cells which suggests that, although non-dividing cells can accommodate L1 retrotransposition [12,89], the cell cycle may promote efficient retrotransposition [90–92]. The mechanism through which the L1 RNP enters the nucleus is not well understood. However, live-cell imaging experiments tracking an L1 reporter construct during cell cycle progression indicate that L1 enters the nucleus as the cell starts to divide, presumably facilitated by nuclear envelope disassembly during mitosis [92].

Another explanation for L1 activity in proliferating NPCs may be a more relaxed chromatin state. A relaxed chromatin state is correlated with pluripotency and proliferation, and differentiated cells have more condensed chromatin than progenitor cells [93]. Open chromatin has been assumed to facilitate L1 activation. By using a transcription activator-like (TAL) effector to target different parts of a mouse L1, which was then fused with a transcriptional activator domain, a recent study demonstrated that L1 transcription directly impacts global chromatin accessibility in the early mouse embryo [94]. L1 transcriptional activity to maintain chromatin openness may be a prerequisite for normal development of the early embryo [94] and it will be interesting to further extend this study into later developmental stages and in the context of neurodevelopment.

Finally, TFs can stimulate L1 to retrotranspose during neuronal differentiation. Sex determining region Y-box 2 (SOX2), which is expressed in stem cells and neural progenitors, and is essential in maintaining a proliferative state, can bind to the 5' UTR of L1 and repress promoter activity [72]. During neuronal differentiation, when SOX2 is downregulated, L1 can become active [72]. Concomitantly, members of the Wnt signalling pathway such as wingless-related integration site family member 3a (WNT3a), which promotes neurogenesis, can also stimulate and increase L1 transcription, providing a window of opportunity for L1 mobilization to occur in differentiating neurons [73]. Hence, the shifting epigenetic and transcriptional landscapes of neurogenesis may provide a unique situation for somatic retrotransposition to occur in the brain.

### 5.2. Retrotransposition in postmitotic neurons

If we accept that endogenous L1 mobilization can occur during adult neurogenesis, can it also occur in postmitotic neurons? This is a fundamental question because the lifespan of mature neurons can be as long as a human lifespan, and therefore, any potential for somatic retrotransposition in this context may lead to the largest absolute accumulation of new L1 insertions found in the brain. Crucially, Kubo *et al*. [89] showed in 2006 that engineered L1 retrotransposition can occur in non-dividing human fibroblasts and hepatocytes and, much more recently, Macia *et al*. [12] confirmed that this was also true for postmitotic neurons. Macia *et al*. compared the retrotransposition of a hybrid adenoviral L1-EGFP vector in dividing versus non-dividing neural cells by infecting NPC cultures with the L1 reporter and concomitantly adding 5'-bromo-2'-deoxyuridine (BrdU), which labels dividing cells. Immunostainings of differentiated NPC cultures infected with L1-EGFP at 31 days post-NPC differentiation showed the presence of neurons expressing only EGFP but not the cycling marker, BrdU. When comparing the number of integrated L1-EGFP copies in cultures infected with the L1 reporter at day 0 (multi-potent NPC) versus day 31 of differentiation (in mature neurons), a sixfold increase in L1-EGFP copies was observed by qPCR against the EGFP splice junction in mature neurons, even when correcting for differences in proliferation rate [12]. This was an astonishing result, as it supports speculation that not only do mature neurons support retrotransposition, but also retrotransposition occurs at even higher rates in neurons than in proliferating neural progenitors. The study relied heavily on qPCR detection of EGFP copies, and used an *in vitro* assay to compare dividing versus non-dividing neural cells. Going forward, it will be interesting to address this question *in vivo*, perhaps by using an inducible knock-in reporter model. Furthermore, it would be very interesting to explore in detail the mechanisms through which retrotransposons escape epigenetic silencing and mobilize in postmitotic neurons.

## 6. Is retrotransposition necessary for healthy brain function?

It is not established whether retrotransposition in the neural genome is part of normal brain function. It is however plausible that, if retrotransposons did contribute to normal brain function, it would be via altered splicing and DNA methylation of genes, with these being routes to perturb the transcriptional output of cells. For example, pseudogene transcripts can carry microRNA (miRNA) recognition sites and facilitate degradation of their target miRNAs, acting as competitive miRNA targets to the miRNA targets of their parent genes [95]. Such pseudogenes carrying miRNA recognition sites are present in temporal lobe neurons, where they may play an important role in regulating the expression of miRNAs [96]. Somatic pseudogene insertions in the brain, if found, may therefore regulate gene regulation as miRNA ‘sponges’ [97]. In another example, experiments using L1-EGFP transgenic rodents, which were either sedentary or allowed to voluntarily run, demonstrated that exercise increased the total number of newborn hippocampal neurons, which correlated with an increase in engineered L1 retrotransposition [98]. However, it remains unclear whether the increase in GFP was due to a greater number of insertions in the running mice or an activation of GFP expression from previously silenced L1-EGFP insertions, due to changes in the epigenetic landscape. Nevertheless, as the hippocampus is an important area for brain structural plasticity, being involved in learning, memory as well as stress regulation, retrotransposition in hippocampal neurons could potentially contribute to neuronal plasticity. Whether retrotransposon insertions can actively contribute to neuronal physiology, and thus impact behaviour, is still debatable, as to date no functional analyses have been published in this area.

## 7. Retrotransposition-induced genomic alterations in neuronal genes and their functional consequences

Retrotransposon insertions can significantly alter protein-coding and regulatory regions of the genome, and thereby affect gene expression and other cellular outputs [63,68,99–103]. L1 insertions can occur within neuronal genes, and therefore have the potential to change the structure of genes or how highly they are expressed [4,7,10,72,99]. The consequences of an intragenic L1 insertion for a cell depend on: (i) the characteristics of the insertion (full-length or 5' truncated L1, sense or antisense to the gene; internally inverted and deleted); (ii) the locus where it integrates; and (iii) the ability of the host cell to silence or compensate for the effects of the L1 insertion [104,105]. The various routes by which L1 insertions can lead to gene expression changes have been reviewed previously [106–108]. L1 insertions can delete sequences at the target site [52,109]. In some cases, these deletions can be quite large, such as the 46 kb deletion reported for the *PDHX* gene encoding pyruvate dehydrogenase, which results in pyruvate dehydrogenase deficiency, a neurodegenerative disorder [110]. Genomic sequences flanking the source L1 5' or 3' end can be transduced along with the L1 to the new integration site, potentially leading to exon shuffling and new genes [111]. Recombination can occur between retrotransposons, causing deletions, duplications or rearrangements in genes [112]. Transcriptional stop sites and polyadenylation signals can be introduced by new retrotransposon sequences, leading to premature transcriptional termination [99,113]. An antisense promoter located in the L1 5' UTR can also create new transcription start sites for genes upstream of the L1 [35,114–116], meaning that both intragenic and intergenic L1 insertions may alter gene expression.

New L1 insertions can lead to aberrant transcriptional splicing [117–121]. Two prominent examples of this are provided by mice bearing spontaneous mutations with neurological phenotypes: the Spastic and Orleans reeler mice. Firstly, the Spastic mouse harbours a germline full-length L1 insertion into a non-coding region of the glycine neurotransmitter receptor β (*Glyrβ*) gene which leads to aberrant splicing of the pre-mRNA by exon skipping, thus resulting in reduced intact subunit β expression in the brain [118,119]. As the β subunit is required for glycine receptor protein assembly, this insertion leads to a decrease in glycine receptors in the brain and a complex motor deficit phenotype. Secondly, the Orleans reeler mouse incorporates a full-length L1 insertion into a coding region of the Reelin (*Reln*) gene, which also leads to exon skipping and a frame shift, in this case generating a 220 bp deletion of the *Reln* mRNA, and leading to inefficient secretion of Reln truncated protein [120,122,123]. As Reln is essential for neuronal migration and cortical lamination, deficiency in its secretion leads to a severe impairment of neuronal migration and, as a consequence, cortical and cerebellar delamination. Subsequent neurological symptoms in *Reln* mutant mice recapitulate the phenotype seen in patients with lissencephaly caused by other *Reln* mutations [124]. Hence, de novo L1 insertions arising in the germline can alter genes expressed in the brain and cause a neurological phenotype, suggesting that similar mutations arising in neurons have the potential to generate a functional change.

## 8. Retrotransposon regulation and environmental factors impacting retrotransposition

### 8.1. Locus-specific regulation of long interspersed element 1 retrotransposition

Retrotransposition depends on a mobile element's intrinsic ability to ‘jump’, and its host cell's capacity to defeat this mobilization. Despite their significant presence in the genome, almost all human L1 copies are 5' truncated, or contain internal mutations and rearrangements, and are not able to mobilize. A few L1 copies are full length, remain transcriptionally active and have intact ORFs and therefore have the capacity to act as source, or donor, elements in a cycle of retrotransposition. An even smaller subset (approx. 6 L1s per individual genome) retrotranspose efficiently *in vitro* when tagged with a fluorescent or selectable marker [50,70] and are referred to as ‘hot’ donor L1s [41,42], although retrotransposition efficiency can vary significantly in different cell types. Hot L1s tend to be the most recently acquired L1 copies in the genome, have very low sequence divergence from the L1 consensus sequence and are polymorphic in the population (found in some individuals but absent in others) [41]. If a cell type supports a high L1 retrotransposition rate, is this due to the activity of a few hot donor L1s, or is it the result of L1 mRNAs being widely expressed in line with concerted, genome-wide dysregulation? To address this question, Philippe *et al*. [105] recently designed an assay that discriminated near-identical donor L1s by mapping the 5' and 3' genome junction sequences of all of the potentially active donor L1s in a set of somatic cell lines and intersecting these data with transcriptional and epigenetic signatures. The authors found that donor L1s were generally inactive, with a limited number active in each of the cell lines examined. These results suggested cell-type-specific activity of retrotransposition-competent L1s, presumably as only some L1s escaped silencing in each cell type. However, a previous study [125] identified a large number of L1s that were actively expressed in multiple hESC lines, suggesting that patterns of donor L1 activation may differ in pluripotent and differentiated cells, albeit with a different approach in each study.

### 8.2. Retrotransposon silencing mechanisms

As retrotransposition events can cause such large effects on gene expression, cell function and ultimately organism fitness, several mechanisms exist to limit and regulate L1 mobilization in germ and neural cells. These mechanisms have been reviewed comprehensively [126,127]. We will focus here on mechanisms thought to regulate retrotransposition during neural cell differentiation, a process that must be tightly regulated during critical stages of brain development, as differences have been observed in retrotransposition efficiency between proliferating NPCs and postmitotic neurons [12,76]. It is known that different DNA methylation patterns are present across neurodevelopmental stages [128]. Presumably, dynamic methylation during developmental processes, such as neuronal differentiation, can offer windows of opportunity for retrotransposition to occur [76]. Additionally, different neuronal subtypes (such as GABAergic interneurons and glutamatergic projection neurons from the prefrontal cortex (PFC)) are known to present differences in their methylation patterns [129]. A recent study investigating methylation of the L1 5' UTR, which is a critical predictor of L1 promotor activity [76,130], found no differences in the levels of methyl-cytosine and 5-hydroxy-methyl-cytosine across frontal cortex, hippocampus, cerebellum and basal ganglia regions in the adult mouse brain [131]. Nevertheless, subtle differences in L1 methylation state across neuronal types and brain areas could potentially be missed when analysing bulk tissue samples.

Several epigenetic factors are known to repress L1. For example, methyl-CpG-binding protein 2 (MeCP2) is present ubiquitously in the body and in higher abundance in mature neurons. MeCP2 binds 5-methyl-cytosine residues in CpG dinucleotides and interacts with histone deacetylase protein (HDAC) and transcriptional SIN3A corepressor complexes to repress transcription [132–134]. MeCP2 knockout mice show an increase in L1 promoter activity consistent with MeCP2 functioning as an L1 repressor [74,135]. Valproic acid inhibition of HDAC1 enhances the transcriptional activity of L1 [136], indicating that HDAC1 is also involved in L1 repression. Another deacetylase suggested to inhibit L1 is mono-ADP ribosyltransferase enzyme, or Sirtuin 6 (SIRT6), that was shown to localize to the L1 promoter [137]. SIRT6 is expressed in neurons and appears to be displaced in oxidative stress conditions and during ageing [138]. Although L1 is repressed by these epigenetic mechanisms, the brain appears to exhibit lower L1 methylation than found for other tissues, such as skin [76].

In addition to epigenetic regulation, retrotransposon activity can be regulated by specific TFs, such as yin yang 1 (YY1), runt-related transcription factor 3 (RUNX3), SOX2 and tumour protein p53 (p53). YY1 is a zinc finger protein expressed ubiquitously in the brain. It is critical for regional patterning of the brain and neuronal differentiation, as it regulates genes including Otx2 and Engrailed 2 [139–141]. YY1 positively regulates L1 by directing the RNA polymerase II complex to its proper binding site [142,143]. RUNX3, which is involved in neurogenesis, development and survival of proprioceptive neurons, also positively regulates the L1 promoter [144–146]. Conversely, the TF SOX2, involved in the maintenance of the multi-potent state of neural stem cells (NSCs), has been shown to inhibit L1 transcription [72,73,76,147–149]. This is also the case for p53, a master regulator of cell cycling that is involved in neural proliferation and differentiation (reviewed in [150]). p53 may suppress L1 retrotransposition through its involvement in H3K9 trimethylation (H3K9me3), a silencing marker, at the L1 5' UTR [151,152]*.* As new L1 insertions carry TF binding sites and potentially attract epigenetic suppression [153], the integration of an L1 into an intron or an intergenic region immediately upstream of a protein-coding gene can alter the expression pattern of that gene. L1 insertions are thought to be particularly harmful if oriented in sense to the gene, based on a depletion of these insertions from the human population [99,154], and also the length of the insertion may influence its impact [99]. Host factors, such as apolipoprotein B mRNA editing enzyme catalytic subunit 3A (APOBEC3A) [155], or DNA repair factors such as ATM [54], can therefore counteract the impact of L1 at the insertion site by limiting the length of L1 insertions [156]. Hence, even if the host cell fails to prevent the integration of a new L1 copy, it may mitigate the potential consequences that L1 insertion has upon the genome.

### 8.3. Environmental factors impacting retrotransposition

Numerous studies have proposed that environmental factors may trigger hyperactivation of L1 and other retrotransposons. However, most of these studies are preliminary and the experiments have been largely performed on cell lines. For instance, heavy metals such as mercury, nickel and cadmium seem to increase L1 retrotransposition in HeLa and neuroblastoma cells [157–159], and so does oxidative stress [160]. Pollutants such as benzo[a]pyrene (BaP), a polycyclic aromatic hydrocarbon ubiquitously found in grilled meats, tobacco smoke, vehicle exhaust, domestic wood and coal fires, have also been found to be involved in L1 retrotransposition [161]. Similarly, an L1-lacZ retrotransposition assay showed that UV light and heat-shock induces an increase in β-galactosidase activity in tumour cell lines [162]. The increase in L1 activity as a result of environmental insults may occur via an indirect pathway, for example, L1 retrotransposon may leverage the DNA double-stranded breaks induced by heavy metal toxicity to mobilize and insert at new genomic locations. Indeed, Morrish *et al*. [163] described an endonuclease-deficient engineered L1 that could readily retrotranspose in cell lines lacking DNA repair mechanisms. However, Farkash *et al*. [164] reported no increase in retrotransposition of an endonuclease-deficient L1 after application of gamma radiation, which is known to induce DNA double-strand breaks. It therefore remains unclear whether L1 can easily integrate, through an endonuclease-independent mechanism, at preformed DNA double-strand breaks. Alternatively, the cellular machinery could use L1 to induce apoptosis or repair DNA damage through a compensatory mechanism [161,163,165]. Although these environmental insults are known to impact neurogenesis and brain function, a link between these factors and an increase in retrotransposition in neurons or brain tissue has not been formally demonstrated *in vivo*.

## 9. Retrotransposition in neurological disease

Retrotransposition in the early embryo and committed germline can result in heritable mutations that cause genetic disease [11,63]. Several retrotransposon insertions involved in genetic neurologic diseases have been reported to date (table 1). As stress-responsive elements, retrotransposons could be activated by an abnormal neural cell environment, and thereby generate somatic insertions with the potential to influence neurological diseases of developmental and degenerative origin. Early developmental stress factors, as well as environmental insults occurring later in life, are major risk factors for developing neuropsychiatric and neurodegenerative disorders [208]. Although many studies report elevated retrotransposon copy numbers, mRNA levels or retroviral markers in neurological diseases, to date there are essentially no confirmed causative links established between any neurological condition and somatic retrotransposition. Here, we present an overview of the most relevant findings in this area from L1 and *Alu* data. The implication of HERVs in neurological disease, such as multiple sclerosis, schizophrenia and bipolar disorder, has been recently reviewed [178,209,210]. The involvement of SVA retrotransposons in neurological disease has also been recently reviewed [211,212]. Processed pseudogenes in the context of neuropathology have not been studied extensively, to our knowledge.

**Table 1.** Studies associating retrotransposons with neurological disease. Here, we consider association as a study having shown that a retrotransposon insertion, or insertions, may cause a disease, or the study having reported elevation of retrotransposon copy number or mRNA level in the brain tissue of affected individuals when compared with healthy individuals.

retrotransposon	neurological disease	
	insertions	activity (mRNA levels, CNV, biomarkers)
HERV	multiple sclerosis [166–168] 3q13.31 microdeletion syndrome [169]	amyotrophic lateral sclerosis [170,171] multiple sclerosis [166–168,172] schizophrenia [173–176] bipolar disorder [173,175,177] HIV-associated dementia [178] major depression [177] autism [179] ADHD [180]
L1	pyruvate dehydrogenase complex deficiency [110] Fukuyama-type congenital muscular dystrophy [181] neurofibromatosis type I [182] ataxia with oculomotor apraxia 2 [183] glioblastoma [184,185] schizophrenia [176] ataxia telangiectasia [54] Coffin Lowry syndrome [186]	major depression [187,188] schizophrenia [187–189] Rett syndrome [74] autism [190] cocaine addiction [191] post-traumatic stress disorder [192]
*Alu*	autosomal dominant optic atrophy [193] adrenoleukodystrophy [194] neurofibromatosis type I [182,195] lipoprotein lipase deficiency [196] spastic paraplegia [197,198] Hunter disease [199] Menkes disease [200] Walker–Warburg syndrome [201] Lesch–Nyhan disease [202] glycogen storage disease type II [203]	post-traumatic stress disorder [192]
SVA	neurofibromatosis type I [204] X-linked dystonia-parkinsonism [101–103,205] Fukuyama muscular dystrophy [206]	n.a.
processed pseudogenes	spinal muscular atrophy [207]	n.a.

### 9.1. Neurodevelopmental and psychiatric disease

A prominent example of a neurodevelopmental disorder that has been linked to retrotransposon activity is Rett syndrome (RTT). RTT is a progressive, severe neurodevelopmental disease associated with a mutation in the *MeCP2* gene [213]. MeCP2 globally regulates methylated DNA and, as mentioned above, has been shown to repress L1 transcription and retrotransposition [74,135]. Mice lacking functional MeCP2 exhibit elevated L1 mRNA expression and L1 copy number in brain [74]. Increased retrotransposition of the L1-EGFP reporter has been observed in *MeCP2* mutant rat NSC cultures and transgenic mice [74]. Elevated L1 mRNA abundance and L1-EGFP reporter activity were also found in NPCs differentiated from induced pluripotent stem cells (iPSCs) derived from RTT patient fibroblasts [74]. However, it remains unclear whether susceptibility to increased L1 retrotransposition in RTT patients can act as a driving force for disease phenotype or progression, or is a peripheral outcome of *MeCP2* mutation.

Psychiatric diseases, such as autism, schizophrenia and bipolar disorder, have also been linked with retrotransposon activity [177,179,187,188,190–192]. The aetiology of these disorders involves polygenic and environmental risk factors. Recreational drugs that cause addiction, such as methamphetamines (METH) and cocaine, are major risk factors for the development of psychiatric disorders [214,215] and have been reported to affect L1 retrotransposition in cell lines and rodent brains [216,217]. One study reported elevated L1 mRNA and ORF2p levels after METH exposure in rat neurogenic areas [217]. Both METH and cocaine can induce L1 retrotransposition in cultured neuroblastoma cell lines, as determined by L1-EGFP [217] and L1-neomycin reporter assays [216]. In cell culture, METH and cocaine do not induce DNA breaks, as revealed by the absence of gamma H2A histone family X (H2AX) phosphorylation at serine 139, a marker for double-strand DNA breaks [216]. Instead, L1 mobilization in response to METH and cocaine treatment may occur via the cyclic AMP response element-binding protein pathway mediating ORF1p access to chromatin [216]. In a recent study, Doyle *et al*. [191] sought to further test the potential link between L1 insertions and cocaine in brain tissue of cocaine addicts and controls. The authors employed a TaqMan ddPCR assay and found no significant increase in the L1 mRNA transcript levels in the cocaine group [191]. WGS performed on pools of neuronal and non-neuronal nuclei, as well as blood samples from cases and controls, revealed no significant increase in the number of L1 insertions in cocaine samples compared with controls [191]. The authors reported gene ontology terms, potentially important for cocaine signalling pathways, to be enriched for genes harbouring L1 insertions found in the cocaine group and not observed in the control. However, the study had several caveats which could be addressed in future research. Samples were pooled across control or case individuals, relying on a ddPCR assay to determine the allele frequency of insertions in each individual. Furthermore, insertions were detected based only on the 3' end of L1 and 100 nt single-end Illumina reads, and therefore were not fully resolved and characterized. How and whether these new insertions ultimately contribute to addiction is still very much unclear. The relevant studies have to date not incorporated single-cell genomic analysis with full characterization of candidate somatic insertions.

Increased retrotransposon copy number and mRNA levels have been reported in schizophrenia and depression [187,192]. Bundo *et al*. [187] revealed a significant increase in L1 ORF2 copy number in neurons isolated from schizophrenia PFC when compared with matched control neurons. This result was replicated in iPSC-derived neurons from patients affected by 22q11 deletion, a mutation found in a number of schizophrenia patients and considered a high genetic risk factor [218]. A consistent increase in L1 copy number was also observed in the PFC tissue of two established schizophrenia animal models (i.e. maternal immune activation induced by polyinosinic : polycytidylic acid (PolyI : C) and epidermal growth factor (EGF)) [187]. WGS performed on neuronal nuclei and liver samples revealed no increase in L1 insertion number in patients when compared with controls. The authors reported that the L1 insertions in schizophrenia patients were more frequently present in genes important for synaptic function and schizophrenia-related genes [187]. However, the insertions were not validated and characterized. Moreover, many of the brain-specific insertions appeared from inactive, old L1 subfamilies, which could not have accounted for de novo retrotransposition. Another study reported significant L1 promotor hypomethylation in the PolyI : C model of maternal immune activation, which could explain elevated L1 transcription [219]. Very recently, Bedrosian *et al*. [79] proposed that low maternal care, another risk factor for schizophrenia and other psychiatric disorders, may elevate L1 retrotransposition in the hippocampus. This study, based largely on a qPCR L1 CNV assay, estimated higher L1 copy number and transcript levels in mice after low maternal care when compared with those reared in high maternal care conditions. The authors reported no change in neurogenesis between the two groups. However, the elevated L1 transcript levels correlated with hypomethylation of L1 at a YY1 TF binding site [79]. As a qPCR assay cannot prove L1 integration, it remains unclear whether the difference in L1 copy number and transcript abundance was reflected by an increase in retrotransposition. Consistent L1 upregulation across various schizophrenia experimental systems indicates a strong association between disease phenotype and L1 activity, although it remains unclear whether L1 is a driver or a passenger with respect to neuropathology and, again, single-cell genomic analyses supporting elevated L1 mobilization, as opposed to L1 CNV, have yet to be published.

Even if elevated somatic retrotransposition ultimately is not found to occur in a given neurological condition, a molecular signature of L1 regulatory disruption may indicate a wider landscape of epigenome abnormality, and prove useful as a diagnostic tool. For example, altered L1 and *Alu* epigenetic regulation has been reported in post-traumatic stress disorder (PTSD), an anxiety condition characterized by persistent re-experiences of a past traumatic event or events [192]. PTSD patients within a cohort of military personnel assessed post-deployment showed hypermethylation of L1 and *Alu* in serum when compared with healthy individuals [192]. Non-PTSD post-deployment individuals also exhibited a hypermethylation pattern when compared with the same individuals pre-deployment. These two observations are intriguing and might suggest an adaptive stress response mediated by retrotransposon repression or pre-existing methylation in PTSD cases compared with controls [220]. These findings could be buttressed by experiments performed using animal models where L1 methylation and CNV could be analysed in brain tissue. Overall, a theme has emerged of L1 activation in a wide range of neurological disorders. More research is required in this area to understand whether there is a functional consequence of L1 mobilization in schizophrenia and other psychiatric disorders.

### 9.2. Neurodegeneration

Neurodegenerative conditions, such as Parkinson's disease, Huntington's disease and Alzheimer's disease, represent a group of nervous system disorders characterized by the selective death of neuronal subsets in particular brain regions [221]. Experimental data showing somatic retrotransposition, or even the broad involvement of retrotransposons, in neurodegeneration are quite scarce. To speculate, dysfunctional epigenetic silencing, deficient DNA repair and oxidative stress could all lead to retrotransposon activation associated with neuronal degeneration or to the progression of degenerative processes. As an instructive example, L1 copy number was found via a qPCR-based assay to be elevated in hippocampal neurons from ataxia telangiectasia patients [54]. These patients presented a loss of function mutation in the *ATM* gene encoding a serine/threonine kinase [222], which leads to neuronal degeneration [223]. ATM responds to double-stranded DNA breaks by phosphorylating downstream factors and activating DNA damage checkpoints, thus leading to cell cycle arrest and to the repair of damaged DNA or p53-mediated apoptosis [223]. *ATM* mutation in cultured human NPCs and *ATM* knockout in L1-EGFP mice significantly increased L1-EGFP retrotransposition, measured by the number of GFP-positive cells, without affecting L1 promoter activity or endogenous ORF1p levels, and with no change in cell division rates or survival [54]. Notably, longer L1 insertions appeared to occur in *ATM* mutant cells [54], potentially owing to the role of ATM in cellular DNA repair, which may interfere with host defences against L1 retrotransposition under wild-type conditions. Further analysis, preferably *in vivo*, is required to corroborate these results, and identify the role of ATM in controlling L1 integration.

As many neurodegenerative conditions are associated with ageing, retrotransposition in neurodegenerative conditions may, in general, reflect conditions encountered in senescent cells. Accumulation of oxidative DNA damage and unrepaired DNA, as well as changes in methylation patterns commonly associated with ageing, might lead to reduced silencing of retrotransposition in the brain. As an example of this, TAR DNA-binding protein 43 (TDP-43) is a nucleic acid binding protein involved in transcriptional repression and RNA metabolism during stress response. Mutant TDP-43 is usually associated with neurodegenerative disorders, but it can also be present in healthy elderly people [224]. Accumulation of TDP-43 in tau-negative and ubiquitin-positive cytoplasmic inclusions is a neuropathological hallmark in neurodegenerative conditions, such as amyotrophic lateral sclerosis (ALS) and frontotemporal lobar dementia (FTLD) [225]. Transcriptomic analyses performed on brain tissue obtained from TDP-43 mutant mice and mice expressing human mutant TDP-43 showed an overall increase in retrotransposon expression [226]. Moreover, surveys of protein–RNA interactions and gene expression performed on FTLD brain samples versus matched controls found a significantly reduced association of mutant TDP-43 at its target retrotransposons in patients [226]. Another recent study, wherein human TDP-43 was expressed in *Drosophila* brains, recapitulated these results from patients and mouse models, demonstrating that TDP-43 dysfunction results in de-repression of retrotransposons [227]. Interestingly, the expression of human TDP-43 specifically in fly glial cells led to an early and significant increase in *gypsy* ERV transcript levels as previously reported to be elevated in ageing flies [228]. Human TDP-43 in the fly glia resulted in a high number of apoptotic nuclei, very significant motor impairment and shortened lifespan. Knockdown of *gypsy* ameliorated this phenotype, suggesting that *gypsy* may be involved in cell death, potentially by mediating DNA damage [227].

Many neurodegenerative disorders are strongly linked with mitochondrial dysfunction. In intriguing recent work, Larsen *et al*. [229] put forward the hypothesis that retrotransposons were involved in mitochondrial gene dysfunction observed in neurodegeneration. Analysis of the retrotransposon sequence content in over a thousand mitochondrial genes, in conjunction with randomly selected protein-coding genes, showed an enrichment for *Alu* within and adjacent to mitochondrial genes [229]. Previous studies have identified L1 and *Alu* retrotransposon insertions in a number of translocase of outer mitochondrial membrane genes [4,230,231]. Moreover, *Alu* insertions in two genes involved in mitochondria stabilization and function led to disorders with a neurodegenerative component [193,194]. More studies, in cell culture and animal models, are required to test if *Alu* activity can preferentially insert into and induce mitochondrial gene disruption.

## 10. Concluding remarks

It is now well established that retrotransposon-driven mosaicism can occur in the mammalian brain. Dynamism in TF activity, and that of other regulatory mechanisms described to act during embryonic and adult neurogenesis, provides a temporal and spatial niche amenable to retrotransposition. Additionally, retrotransposition may not be limited to NPCs; it may also take place in postmitotic neurons. Further proof is required to be certain that this can occur *in vivo*, and the regulatory mechanisms involved in allowing L1 retrotransposition in mature neurons require further investigation. Environmental stress, as well as genetic dysfunction in L1 silencing factors, can result in high retrotransposition levels, and possibly lead to neurological disease. This also requires extensive investigation, as we lack even basic understanding of the pathways on which stress factors or dysfunction in silencing factors can act to impact retrotransposition in neural cells. Our understanding of retrotransposition in the brain is still very much in its infancy.

Further advances in single-cell genomics (and perhaps taking Eric Kandel's reductionist approach of ‘one cell at a time’ to focus on very well-defined neuronal subtypes [232]) to understand the precise timing of retrotransposition, and its regulation, are required at this point, and will also advance our knowledge of which neuronal types and contexts best support endogenous L1 retrotransposition. Genome-editing tools, such as the CRISPR–Cas9 system [233], may be employed in the near future to resolve L1 insertions identified in patient samples and introduce these faithfully into neuronal cultures or animal models, to evaluate their impact on gene expression and, potentially, neurobiological phenotype. This approach may prove to be a viable alternative to the current transgenic approaches used to study engineered L1 mobilization in the brain, and would bring us closer to understanding whether endogenous retrotransposition has an impact upon neurobiology and neurological disease.

## Data accessibility

This article has no additional data.
